# Matrix Stiffness Affects Glycocalyx Expression in Cultured Endothelial Cells

**DOI:** 10.3389/fcell.2021.731666

**Published:** 2021-10-07

**Authors:** Marwa Mahmoud, Limary Cancel, John M. Tarbell

**Affiliations:** Tarbell Lab, Department of Biomedical Engineering, The City University of New York, New York, NY, United States

**Keywords:** endothelial cells, matrix stiffness, glycocalyx, heparan sulfate, glypican 1

## Abstract

**Rationale:** The endothelial cell glycocalyx (GCX) is a mechanosensor that plays a key role in protecting against vascular diseases. We have previously shown that age/disease mediated matrix stiffness inhibits the glycocalyx glycosaminoglycan heparan sulfate and its core protein Glypican 1 in human umbilical vein endothelial cells, rat fat pad endothelial cells and in a mouse model of age-mediated stiffness. Glypican 1 inhibition resulted in enhanced endothelial cell dysfunction. Endothelial cell culture typically occurs on stiff matrices such as plastic or glass. For the study of the endothelial GCX specifically it is important to culture cells on soft matrices to preserve GCX expression. To test the generality of this statement, we hypothesized that stiff matrices inhibit GCX expression and consequently endothelial cell function in additional cell types: bovine aortic endothelial cells, mouse aortic endothelial cell and mouse brain endothelial cells.

**Methods and Results:** All cell types cultured on glass showed reduced GCX heparan sulfate expression compared to cells cultured on either soft polyacrylamide (PA) gels of a substrate stiffness of 2.5 kPa (mimicking the stiffness of young, healthy arteries) or on either stiff gels 10 kPa (mimicking the stiffness of old, diseased arteries). Specific cell types showed reduced expression of GCX protein Glypican 1 (4 of 5 cell types) and hyaluronic acid (2 of 5 cell types) on glass vs soft gels.

**Conclusion:** Matrix stiffness affects GCX expression in endothelial cells. Therefore, the study of the endothelial glycocalyx on stiff matrices (glass/plastic) is not recommended for specific cell types.

## Introduction

Endothelial cells (ECs) are highly sensitive to mechanical forces and respond to these forces by altering gene expression and downstream signaling pathways (Davies, 2009; Baeyens et al., 2016; Chistiakov et al., 2017). Age/disease related arterial stiffness along with blood flow shear stress are prominent mechanical forces controlling endothelial cell function and vascular disease (Duprez, 2010; Mitchell et al., 2010; Maruhashi et al., 2018).

Endothelial cells lining the vasculature are covered by a multifunctional surface layer of glycans that is referred to as the endothelial glycocalyx (GCX). The common classes of glycans found in the GCX are glycoproteins and proteoglycans. Heparan sulfate proteoglycans (HSPG) are composed of core proteins, including Glypicans that are bound to the plasma membrane by GPI anchors and Syndecans that are transmembrane, with covalently bound glycosaminoglycan (GAG) chains. Heparan sulfate (HS, GAG associated with Glypicans and Syndecans), chondroitin sulfate (CS, GAG associated with Syndecans) and Hyaluronic acid (HA, non-sulfated GAG that binds to surface receptors, e.g., CD44) are the prevalent GAGs on EC surfaces (Weinbaum et al., 2007; Tarbell and Cancel, 2016).

The endothelial GCX is mechanosensor that translates external forces into genetic and functional changes in cells. In addition, the GCX plays an essential role in maintaining EC integrity and vascular homeostasis by preserving barrier function, suppressing inflammation, and cell turnover and mediating flow-induced nitric oxide release (Ebong et al., 2010; Cancel et al., 2016; Tarbell and Cancel, 2016; Bartosch et al., 2017; Mitra et al., 2017; Russell-Puleri et al., 2017). The expression of the endothelial glycocalyx is suppressed under pathological conditions such as hypertension, aging, and disturbed blood flow (Mitra et al., 2017; Harding et al., 2018; Machin et al., 2018; Nelson et al., 2018). We have previously shown using human umbilical vein endothelial cells (HUVECs) and rat fat pad endothelial cells (RFPECs) grown on polyacrylamide (PA) gels of varying stiffnesses representative of healthy or aged/diseased vessels, that age/disease related stiffness promotes endothelial cell inflammation and vascular disease through the suppression of the proteoglycan Glypican 1 and its associated GAG, HS. Glypican 1 in turn plays a protective role in preventing EC dysfunction in response to stiffness (Mahmoud et al., 2021).

We have not considered Perlecan or other matrix proteoglycans that are not bound directly to the EC by a GPI anchor (glypican) or a transmembrane anchor (syndecan) as discussed in Varki et al. (2009). We are only looking at cell bound molecules that might be involved in mechanotransduction. HS and HA were the only GAGs studied in Mahmoud et al. (2021) because in Pahakis et al. (2007) in RFPECs, it was shown that CS was not involved in mechanotransduction to produce nitric oxide (NO) or prostacyclin (PGI_2_). In Zeng et al. (2012) it was shown that CS had much lower surface concentration than either HS or HA. In addition, Syndecan 1 and CD44 were not affected by stiffness on HUVECs and RFPECs in Mahmoud et al. (2021) so they were not included in the present study.

Matrix stiffness is defined as the rigidity of the substrate on which cells grow as typically characterized by the Young’s modulus. Most endothelial cell culture studies involve the seeding of cells on stiff surfaces such as plastic cell culture surfaces or glass. The stiff matrices may have an influence on the expression of the endothelial glycocalyx and thus may impact the study of the glycocalyx *in vitro*. As a follow up to our previous study that utilized HUVECs and RFPECs (Mahmoud et al., 2021), here we examine additional common EC types (BAECs, MAECs, and bEnd3) to examine whether the expression of the glycocalyx protein Glypican 1, and GAG HS are affected by matrix stiffness in different endothelial cell types. Our previous study concluded that reduction of Glypican 1 and HS with increased stiffness were associated with endothelial dysfunction. For that reason, we will focus on studying the expression of Glypican 1 and HS in different commonly used cell types and not on endothelial function. Here we show that matrix stiffness inhibits glycocalyx expression in multiple types of endothelial cells widely used in cell culture studies, suggesting that studies of the glycocalyx on glass and plastic should be interpreted with caution and that softer substrates are encouraged to be employed for studies involving glycocalyx biology.

## Materials and Methods

### Culture of Cells on to Polyacrylamide Gels

Bovine Arterial Endothelial Cells (BAECs), Mouse Aortic Endothelial Cells (MAECs), and Brain-derived endothelial cells 3 (bEnd3) cells were maintained and cultured as previously described (Cancel et al., 2007, 2021; Nikmanesh et al., 2019). BAECs and MAECs were used at passages P5–7, and bEnd3 at P 24–26. Cells were cultured on Polyacrylamide gels as described previously (Mahmoud et al., 2021). Briefly, PA gels were synthesized using different percentages of 30% Acrylamide (Sigma) and Bis-Acrylamide (Sigma) solutions to give a substrate stiffness of either 2.5 or 10 kPa. Cells were seeded on to glass coverslips covered with Fibronectin (Sigma) (60 μg/ml)-coated Polyacrylamide gels and placed into 6-well plate cell culture dishes (Corning). Alternatively, Cells were seeded directly onto Fibronectin-coated glass coverslips for the glass control. Following 48 h of seeding, the cells were processed for immunostaining or for lysis followed by mRNA isolation as previously described (Mahmoud et al., 2021).

### Quantitative Polymerase Chain Reaction

Following mRNA isolation, reverse transcription was carried out using High-Capacity cDNA Reverse Transcription Kit (ThermoScientific), following the manufacturer’s instructions. The reaction took place using a thermal cycler (DNA Engine, Biorad) and thermal profile was determined following the manufacturer’s recommendations. Gene expression was assessed by quantitative polymerase chain reaction (qPCR) on an ABI Prism 7000 Sequence detection system, using gene-specific primers for Glypican 1 and housekeeping gene HPRT (Mahmoud et al., 2021). Data was analyzed using the comparative Ct (2^–ΔΔCT^) method.

### Immunostaining

The expression of GAGs was assessed in ECs by immunostaining. Heparan Sulfate and Hyaluronic Acid expression were assessed as described previously (Mahmoud et al., 2021). The cells were briefly washed with 1 × Dulbecco’s phosphate-buffered saline (DPBS) and fixed with 2% paraformaldehyde/0.1% glutaraldehyde for 30 min at RT, then blocked with 2% Goat serum (GS, Invitrogen, United States) for 30 min. This was followed by an overnight incubation at 4°C with mouse monoclonal primary antibody (For HS;1:100; 10E4 epitope, AMS Biotechnology, United States; or HepSS-1, US Biological, United States or for HA; biotinylated hyaluronic acid binding protein (HABP; 50 μg/mL; EMD Chemicals, United States). Cells were then washed three times in 1 × DPBS, and finally incubated with Alexa Fluor 488 goat anti-mouse secondary antibody for HS (1:400; Molecular Probes, United States) or for HA (Alexa Fluor 488 anti-Biotin (1:100; Jackson ImmunoResearch Lab, United States) for 30 min at RT followed by three washes in 1 × DPBS. The cells were visualized by confocal laser imaging using a ZEISS LSM 800 microscope. Image collection and analysis were carried out as in Zeng et al. (2012). Briefly, Z stacks were obtained with interval slices of 0.4 μm. Images were analyzed using Image J software (1.47 V). The maximum intensity Z-projection of the green Z-series stack (Alexa Fluor 488 channel) was created from which intensity and coverage were calculated.

### Statistical Analysis

All results were generated from at least three independent experiments. Differences between samples were analyzed using a One-way ANOVA for comparison among more than two groups (^∗^*p* < 0.05, ^∗∗^*p* < 0.01, ^∗∗∗^*p* < 0.001). The mean + Standard Error of mean (SEM) was plotted in all graphs.

## Results

We assessed the effects of matrix stiffness on GCX expression by culturing multiple cell types; BAECs, MAECs, and bEnd3 on polyacrylamide gels at a substrate stiffness of either 2.5 kPa (mimicking the subendothelial layer stiffness of young/healthy arteries) or 10 kPa (mimicking the subendothelial layer stiffness of aged/unhealthy arteries), or on glass (Mahmoud et al., 2021).

**FIGURE 1:**
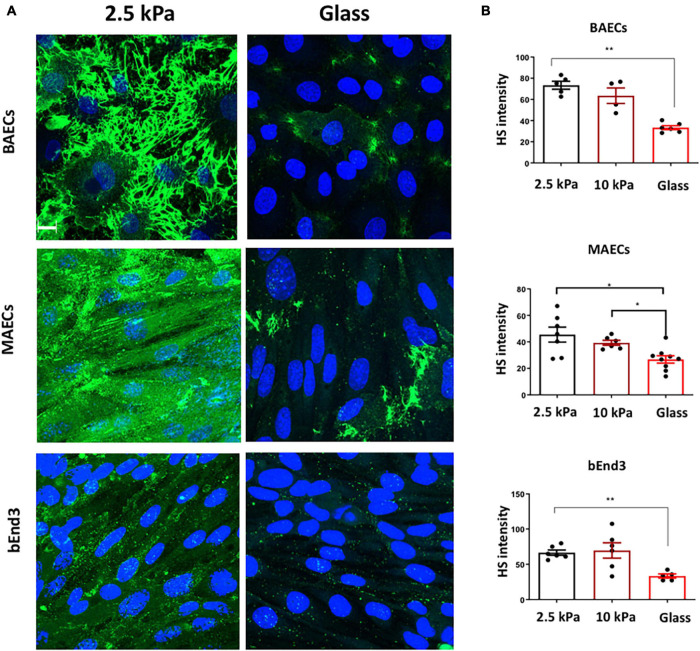
Stiffness inhibits Heparan Sulfate GAG expression on the surface of endothelial cells. **(A)** Bovine aortic endothelial cells (BAECs), Mouse aortic endothelial cells (MAECs) and brain-derived endothelial cells 3 (BEND3) were cultured onto polyacrylamide gels at stiffness of 2.5 kPa (soft gel), 10 kPa (stiff gel) or on glass until confluent. Heparan Sulfate (HS) (green) expression was assessed by immunostaining. Cell nuclei were identified using DAPI 4′,6-diamidino-2-phenylindole (DAPI) (blue). Scale bar = 10 μm. **(B)** Heparan sulfate expression was quantified as fluorescent intensity. Mean values ± SEM are shown. *N* = 6. *P < 0.05 and **P < 0.01 and using a One-way ANOVA test. N.S indicates no statistically significant difference.

Glycocalyx expression was assessed by immunostaining for the most abundant glycocalyx GAG side chain, heparan sulfate (HS). HS expression was inhibited on glass vs on soft, 2.5 kPa, gels for BAECS, MAECS, and bEnd3 cells (Figures 1A,B). These data revealed that similar to HUVECs and RFPECs (Mahmoud et al., 2021) these endothelial cells types also showed reduced HS expression on stiff matrices.

**FIGURE 2:**
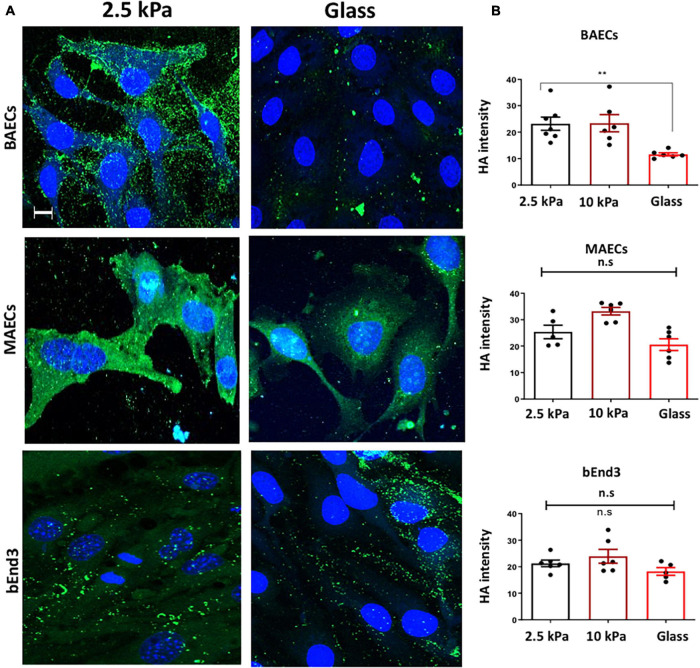
Stiffness inhibits Hyaluronic Acid GAG expression on the surface of BAECs, but not MAECs and BEND3 cells. **(A)** BAECs, MAECs, and BEND3 were cultured onto polyacrylamide gels at stiffness of 2.5 kPa (soft gel), 10 kPa (stiff gel) or on glass until confluent. Hyaluronic Acid (HA) (green) expression was assessed by immunostaining. Cell nuclei were identified using DAPI 4′,6-diamidino-2-phenylindole (DAPI) (blue). Scale bar = 10 μm. **(B)** Hyaluronic Acid expression was quantified as fluorescent intensity. Mean values ± SEM are shown. *N* = 6. *P < 0.05 and **P < 0.01 using a One-way ANOVA test. N.S indicates no statistically significant difference was found.

We next assessed the expression of Hyaluronic Acid (HA) GAG on the surface of endothelial cells. Immunostaining showed that the expression of HA was significantly inhibited on the surface of BAECs, but not on MAECs and bEnd3 cells cultured on glass vs. on soft 2.5 kPa PA gels (Figures 2A,B). These data show that HA expression is not consistently affected in different cell types in response to stiffness. This data is consistent with our previously published data showing that HA expression was not affected by stiffness in HUVECs, whereas in RFPECs HA expression was inhibited by stiff matrices (Mahmoud et al., 2021).

**FIGURE 3:**
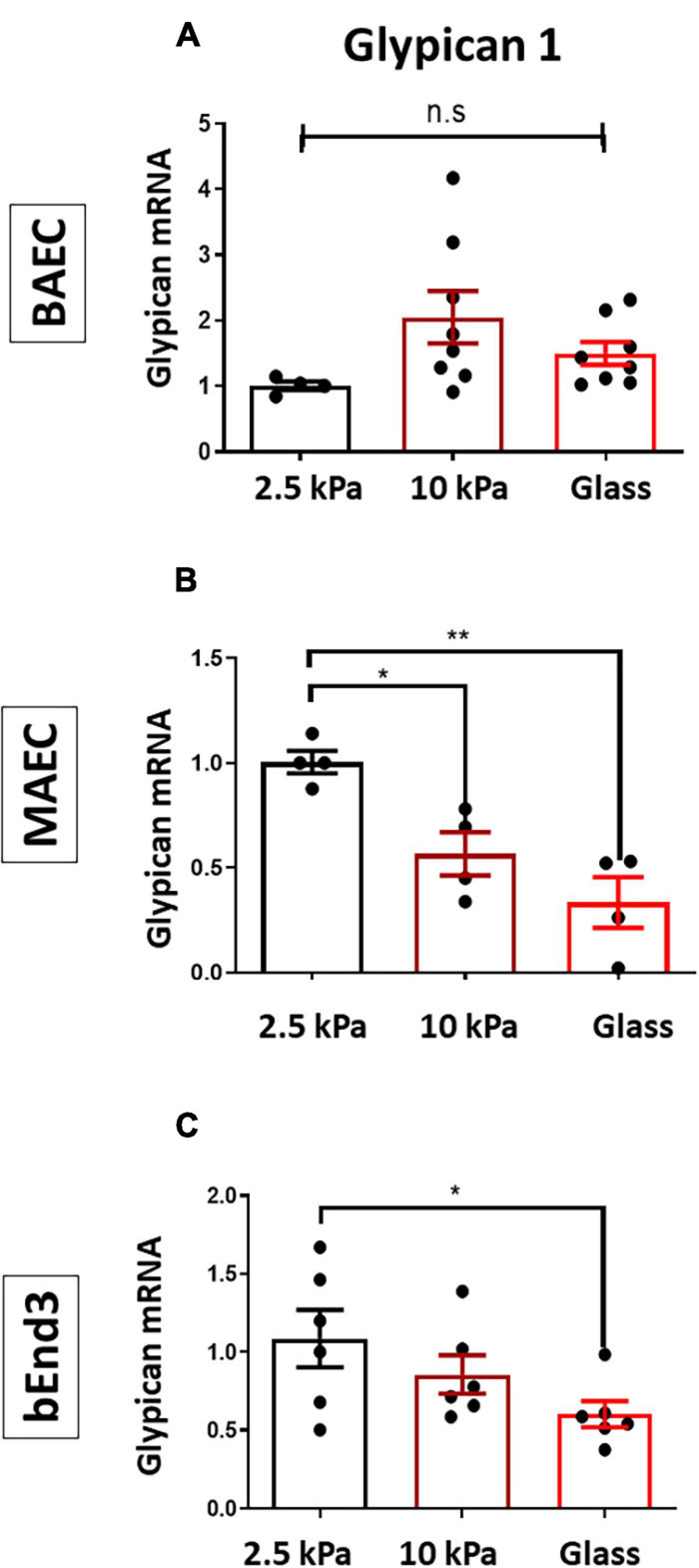
Stiffness inhibits the expression of Glypican 1 in endothelial cells. **(A)** BAECs, **(B)** MAECs, and **(C)** bEnd3 were cultured onto polyacrylamide gels at stiffness of 2.5 kPa (soft gel), 10 kPa (stiff gel) or on glass until confluent. The expression of Glypican 1 mRNA was assessed by qPCR from cells cultured on either 2.5,10 kPa gels or on glass. Hypoxanthine phosphor-ribosyl transferase (HPRT) was used as a housekeeping gene. Mean values ± SEM are shown. *N* = 5–8, *P < 0.05 and **P < 0.01 using a One-way ANOVA test. N.S indicates no statistically significant difference was found.

We have previously shown that matrix stiffness promoted EC dysfunction through inhibiting the GCX proteoglycan Glypican 1 (Mahmoud et al., 2021). Thus, to assess if stiffness also inhibited Glypican 1 in other endothelial cell types we assessed Glypican 1 mRNA expression by qPCR in BAECs, MAECs and bEnd3 cells. The results showed that Glypican 1 expression was inhibited in MAECs and bEnd3 cultured on stiff matrices (glass/10 kPa gels) vs. on soft gels (Figures 3A–C), whereas Glypican 1 expression was not significantly changed in BAECs.

**FIGURE 4:**
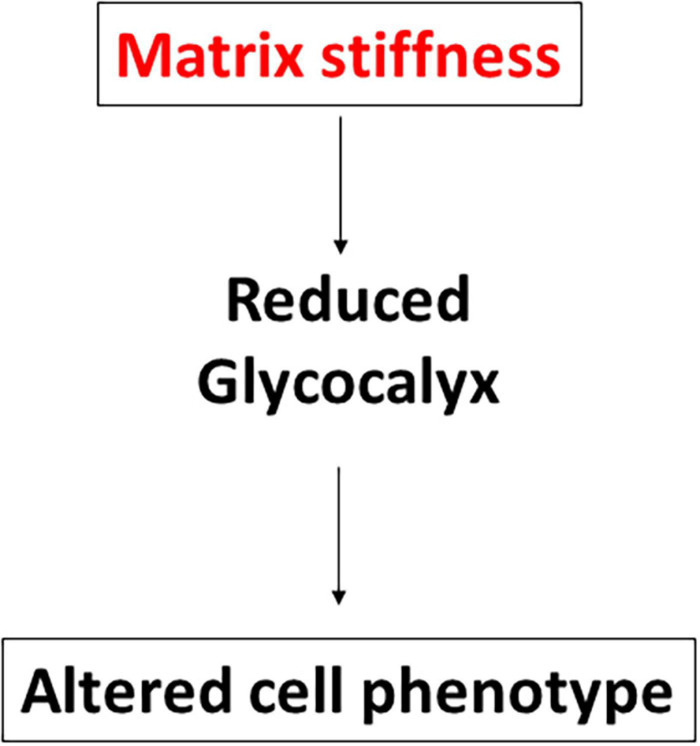
Stiffness inhibits GCX expression in different cell types. Schematic to show that enhanced stiffness inhibits GCX expression through suppressing HS or HA glycosaminoglycan (GAG) expression. Stiffness inhibits the expression of the GCX core protein Glypican 1. The reduction of GCX expression subsequently alters endothelial cell phenotype.

**FIGURE 5:**
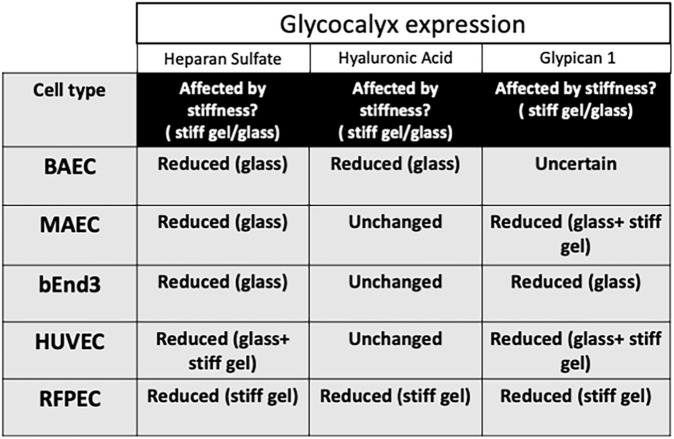
Stiffness inhibits GCX expression in different cell types differently. Summary table to show how matrix stiffness inhibits HS, HA, and Glypican 1 differently for cells cultured on soft, stiff gels or on glass.

The results of these new experiments with BAECs, MAECs, and bEnd3 cells along with previous results for HUVECs and RFPECs are summarized in Figures 4, 5. The data reveal that HS was inhibited in response to matrix stiffness in all cell types tested, however, HA and Glypican 1 expression were affected differently in different cell types. Glypican 1 expression was reduced in all tested cell types except BAECs. The expression of HA was affected in RFPECs and BAECs cultured on stiff matrices (glass/10 kPa gel), but not the other 3 cell types. The data suggest that depending on the endothelial cell origin the endothelial GCX components (HS, HA, and Glypican 1) are inhibited by matrix stiffness. Matrix stiffness inhibition of the endothelial glycocalyx potentially leads to changes in EC function (Figure 4).

## Discussion

With increased age and hypertension, arteries lose elasticity and become thicker, giving rise to arterial stiffness. Arterial stiffness is an underlying risk factor for cardiovascular diseases including atherosclerosis and stroke (Mitchell et al., 2010). Arterial stiffness promotes endothelial cell dysfunction (enhanced inflammation, proliferation, and permeability) driving the progression of cardiovascular diseases (Duprez, 2010; Maruhashi et al., 2018). Although the glycocalyx has not been studied directly in the context of arterial stiffness *in vivo*, our lab has recently shown that age-mediated stiffness directly inhibits the GCX core protein Glypican 1 in a mouse model (Mahmoud et al., 2021). Several studies have indirectly linked the expression of the GCX with arterial stiffness; atheroprone shear stress promotes stiffness and suppresses GCX expression in ECs and is associated with the development of atherosclerosis (Pahakis et al., 2007). GCX expression, including Glypican 1, is inhibited in hypertension (Nelson et al., 2018). Furthermore, in the context of age-mediated stiffness, studies have shown that the expression of GCX was reduced in aged mouse and human subjects vs younger subjects (Machin et al., 2018; Osuka et al., 2018).

Here we showed that the expression of glycocalyx GAGs and core protein Glypican 1 are affected by matrix stiffness in different cell types commonly used in glycocalyx and cell biology research including: HUVECs, BAECs, MAECs, RFPECs, and bEnd3 (Figure 5). We have shown that the expression of HS GAG was inhibited on stiff culture matrices in HUVECs, RFPECs (Mahmoud et al., 2021), BAECs, MAECs, and bEnd3 cells. The expression of HS was consistently inhibited in cells cultured on glass as opposed to stiff gels (10 kPa) which may indicate that the threshold of stiffness that inhibits HS expression is at a value higher than 10 kPa. Interestingly, the expression of HS was inhibited in RFPECs cultured on stiff gels as opposite to glass where the expression of HS was not altered. Fortuitously our lab has frequently studied the glycocalyx in RFPECs cultured on glass (Thi et al., 2004; Cancel et al., 2007; Ebong et al., 2011; Zeng et al., 2012, 2013, 2014; Zeng and Tarbell, 2014; Bartosch et al., 2017, 2021; Mahmoud et al., 2021). In contrast, the expression of HA did not show a consistent change in expression in cells cultured on stiff matrices (10 kPa gel/glass). Of the cell types investigated only BAECs in this study showed a reduction in HA in cells cultured on stiff matrices; this also includes RFPECs as tested previously (Mahmoud et al., 2021). In summary, HS was reduced by stiffness in all five cell types; glypican 1 was reduced in 4 out of 5 cell types tested; and HA was reduced in only 2 of 5 cell types. These observations suggest that HS and glypican 1 that covalently binds HS are most clearly affected by stiffness.

The mRNA expression of the glycocalyx core protein Glypican 1 was suppressed on stiff matrices in MAECs and bEnd3 as shown before in HUVECs and RFPECs (Mahmoud et al., 2021). In addition, the expression of Glypican 1 was also reduced in MAECs *in vivo* in a mouse model of age-mediated stiffness (Mahmoud et al., 2021). Consistent with this, using Glypican 1 gene silencing and gene over expression *in vitro*, along with Glypican 1 KO endothelial cells *in vivo* revealed that the loss of Glypican 1 correlated with enhanced characteristics of endothelial cell dysfunction such as inflammation, endothelial mesenchymal transition (EndMT), and proliferation, along with reduced protective nitric oxide signaling (Mahmoud et al., 2021). Although Syndecan 1 and CD44 were not affected by stiffness in HUVECs and RFPECs, we can’t draw strong conclusions about Syndecan 1 and CD44 in the additional cell types since they were not studied directly.

The observation that expression of different glycocalyx components (HS, HA, and Glypican 1) is affected by substrate stiffness differently depending on the cell type is intriguing. To understand this observation, it will be important to conduct a gene microarray study using different endothelial cell types and to assess a wide array of glycocalyx genes and genes responsible for endothelial dysfunction (inflammation, proliferation, and EndMT). This will determine which subset of genes of the glycocalyx are affected by matrix stiffness and whether they correlate with enhanced expression of genes involved in EC dysfunction.

It could well be that other glycocalyx core proteins such as Syndecans that support HS, along with the CD44 receptor that supports HA are also affected by stiff matrices in certain cell types. However, based on our previous work we have shown in RFPECs and HUVECs that Glypican 1 was the only significantly suppressed protein in response to matrix stiffness out of a group of genes encoding GAG synthesis, core proteins and glycocalyx degradation proteins (Mahmoud et al., 2021). Syndecan1 and CD44 expression were not altered by matrix stiffness in HUVECs and RFPECs (Mahmoud et al., 2021).

There have been reports of a reduced glycocalyx layer in *in vitro* studies which rendered the study of the glycocalyx difficult in some cells (Barker et al., 2004; Potter and Damiano, 2008; Chappell et al., 2009; Janczyk et al., 2010). Our work here provides insight into the optimal conditions to study the endothelial glycocalyx – on soft matrices as opposed to tissue culture plastic or glass. Most *in vitro* studies of the glycocalyx have been carried out under static conditions as we have done in our previous manuscript (Mahmoud et al., 2021). We have also shown that the *in vitro* effects are reciprocated *in vivo* in mouse aortic endothelial cells which have been exposed to flow (Mahmoud et al., 2021). This observation suggests that the effects of stiffness, whether with or without flow, on the reduction of GCX expression is consistent.

The mechanism by which stiffness inhibits the glycocalyx in endothelial cells is not fully known. In our previous work we ruled out the possibility of a degradation mechanism, specifically with regard to Glypican 1, but not HS (Mahmoud et al., 2021). The mechanism could potentially involve the action of miRNAs. Glypican 1 has been shown to be a target of miR-149 in cell types including melanoma cells and endothelial cells (Jin et al., 2011; Chamorro-Jorganes et al., 2014; Lai et al., 2017). In addition, the mechanism could involve signal mechanotransduction from upstream mechanosensing pathways such as integrins and stretch activated ion channels such as Piezo 1. These pathways may result in the suppression of Glypican 1 and GAGs in response to matrix stiffness. Future work will be required to determine the mechanisms by which stiffness inhibits the glycocalyx and whether these mechanisms are conserved in different endothelial cells.

In conclusion, this work revealed that commonly used cell types in cell biology studies are sensitive to substrate stiffness in regard to expression of their cell surface glycocalyx. These observations have implications for our understanding of endothelial cell biology as demonstrated by studies showing that a reduction in glycocalyx expression correlates with changes in EC function (Baeyens et al., 2016; Cancel et al., 2016; Mitra et al., 2017; Bar et al., 2019; Mahmoud et al., 2021). Our previous work has shown that a reduction in Glypican 1 along with its GAG heparan sulfate resulted in enhanced inflammation, proliferation, EndMT and reduced protective nitric oxide signaling in endothelial cells, all characteristics of endothelial cell dysfunction. There have been numerous studies which show that the reduction of the endothelial glycocalyx layer thickness/expression occurs in disease conditions including atherosclerosis, hypertension and in aging (Machin et al., 2018; Nelson et al., 2018; Osuka et al., 2018; Bar et al., 2019). All of these conditions have a common element of enhanced matrix stiffness. An important question to address is whether glycocalyx protection or restoration can rescue endothelial cell integrity and prevent disease progression.

The observations from this work reveal that the culture of endothelial cells on stiff matrices such as glass or tissue culture plastic may give rise to reduced glycocalyx expression and consequential changes in cell function triggering endothelial cell dysfunction. Thus, for the *in vitro* study of the glycocalyx in cultured cells, in order to generate a rich glycocalyx layer, it would be optimal to study the glycocalyx in cells cultured on soft matrices as opposed to tissue culture plastic and glass. Although the use of softer substrates such as polyacrylamide gels (2.5 kPa) may be the most obvious recommendation to be drawn from our study, we did observe that the immortalized RFPEC cell line expressed abundant glycocalyx on glass. Other immortalized cell lines may also preserve the glycocalyx on glass? We hope that our findings allow labs to optimize their methods for studying the endothelial GCX *in vitro.*

## Data Availability Statement

The original contributions presented in the study are included in the article/supplementary material, further inquiries can be directed to the corresponding authors.

## Author Contributions

MM conducted all experiments, gathered the data, interpreted the results, and wrote the manuscript. LC gave useful advice during the experiment discussion. JT interpreted the results, supervised the research, and reviewed the manuscript. All authors contributed to the article and approved the submitted version.

## Conflict of Interest

The authors declare that the research was conducted in the absence of any commercial or financial relationships that could be construed as a potential conflict of interest.

## Publisher’s Note

All claims expressed in this article are solely those of the authors and do not necessarily represent those of their affiliated organizations, or those of the publisher, the editors and the reviewers. Any product that may be evaluated in this article, or claim that may be made by its manufacturer, is not guaranteed or endorsed by the publisher.
